# A Randomized Feasibility Trial of a Fundamental Motor Skill Parent-Mediated Intervention for Children with Autism Spectrum Disorders

**DOI:** 10.3390/ijerph182312398

**Published:** 2021-11-25

**Authors:** Luis Columna, Laura A. Prieto, Pamela Beach, Natalie Russo, John T. Foley

**Affiliations:** 1 Department of Kinesiology, University of Wisconsin at Madison, Madison, WI 53706, USA; lprieto@wisc.edu; 2 Department of Kinesiology, Sport Studies, and Physical Education, State University of New York at Brockport, Brockport, NY 14420, USA; pbeach@brockport.edu; 3 Department of Psychology, Syracuse University, New York, NY 13244, USA; nrusso@syr.edu; 4 Department of Physical Education, State University of New York at Cortland, Cortland, NY 13045, USA; John.Foley@cortland.edu

**Keywords:** autism spectrum disorders, interventions, motor skills, parents, physical activity

## Abstract

The purpose of this pilot study was to examine the feasibility of a fundamental motor skills (FMS) intervention with two groups on the acquisition of FMS of children with autism spectrum disorders (ASD). We randomly assigned families (*n* = 15) of children with ASD aged 4–11 years into two groups (a workshop or a home-based group) focused on FMS development. Both groups participated in a 10-week intervention and were given the same instructional manual and adapted physical activity equipment. The workshop group also attended four in-person workshops targeting the needs of children with ASD and their parents. Children were tested on their FMS using the third edition of the Test of Gross Motor Development at the start and end of the intervention and then three months following the intervention. The recruitment rate was 50%, and the retention rate was 80% for all participants. The intervention for groups was safe and accepted by the participants as evaluated by post-program interviews. The outcomes of this pilot study suggest that parents can facilitate the acquisition of FMS of their children with ASD. Although these results are positive, there is a need to further identify effective interventions for FMS development in children with ASD.

## 1. Introduction

Diagnosed in approximately 1 in every 54 children, autism spectrum disorder (ASD) is one of the most common developmental disabilities in the United States [1]. Researchers have indicated that children with ASD tend to exhibit delays in their fundamental motor skills (FMS) [2,3]. Fundamental motor skills (FMS) are considered building blocks for more complex context-specific skills, such as fielding ground balls, golf drives, and hockey slap shots. FMS, commonly divided into object control (or often referred to as ball skills, e.g., striking, catching, overhand throwing) and locomotor skills (e.g., running, sliding, hopping), do not develop naturally and require additional time and practice [4]. FMS delays are so common in children with ASD that they are often considered a cardinal feature of ASD [5]. As such, in addition to practice, children need specific extrinsic feedback to effectively develop and improve the performance of these skills [6]. However, certain groups of children, including those with ASD, tend to have minimal opportunities to practice FMS and subsequently may lag behind their peers in these basic skills [5]. Furthermore, as children with ASD age, delays in their FMS become more evident than their same-age peers without disabilities [7]. If children with ASD acquire FMS, it may open the doors for them to increase opportunities to participate in sports, dance, and other physical activities [8]. Proficiency in FMS can increase the likelihood of participating in physical activity and the potential to build social skills.

Fortunately, intervention research has demonstrated that children with ASD can improve FMS proficiency [5]. For instance, a study by Bremer et al. [9] found improvements in adaptive skills, object control, and overall motor scores of 4-year-old children with ASD following the completion of a 12-week FMS intervention compared to a control group. Children with ASD aged 3 to 7 years also improved their FMS after two 6-week blocks of an FMS intervention [10], and Ketcheson and colleagues [11] also reported significant improvements after an 8-week FMS intervention. These interventions reveal promising results for improving motor skill competence in children with ASD. Elliott and colleagues [12] examined the secondary effects of an FMS intervention for children aged 4 years old with ASD by interviewing parents. Four themes were identified in addition to motor skill improvement, including social, listening, turn-taking, and transition skills. Benefits were also identified regarding the family unit, such as improved sibling interactions.

It is important to note that parents were not part of these interventions. Typically, behavioral intervention studies have demonstrated that children with ASD rely heavily on parents and caregivers to model effective strategies for behavior and emotion regulation [13]. Unfortunately, when it comes to FMS, parents of children with ASD may lack the knowledge or skills to be able to effectively teach or engage their children in these types of activities [14,15]. Early intervention programs tend to include parents as part of the intervention, but these programs typically focus on improving communication skills with minimal attention to the improvement in FMS [9]. Although children with ASD may participate in physical education at school, the motor skills they learn at school are sometimes not utilized at home and/or other settings, such as community parks [16]. A possible reason for this might be because many teachers who provide FMS instruction to children with ASD are generalists, and many do not have formal training in working with this population [9]. To date, several studies have examined the effectiveness of parent-supported FMS interventions among youth for at-risk preschoolers but have not focused specifically on children with ASD [17,18].

In one study, Hamilton et al. [18] assessed the effect of a combined physical activity professional and parent instruction on object control skills during a one-day workshop in children (ages 3 to 5 years) who were at risk of developmental delays. The results of this study demonstrated that children who received additional parental support (in combination with teacher’s instruction) did better on their motor skills than the students who only received instruction from their teachers. More recently, Altunsöz et al. [17] designed an intervention in which one group of children with developmental delays received instruction in object control skills as part of their head start program. The other group of children received the same instruction, accompanied by parental support at home. Parental home support consisted of providing parents with lesson plans and physical education-related equipment to practice their home activities. The results indicated that both groups improved their locomotor skills in comparison to the control group. However, there were no significant differences between groups with the additional support from parents. While early evidence supports that parents are essential for FMS acquisition, the study by Altunsöz et al. [17] indicated there is still more to research about the specific role parents play in FMS development. However, limited information is available regarding the parental role in the acquisition of FMS of children with ASD.

Recently, attempts have been made to include parents of children with ASD in physical activity interventions. Healy and Marchand [19] designed Project CHASE (Children with Autism Supported to Exercise) as a parent-mediated web-based physical activity intervention. This intervention aimed to examine how parents of children with ASD perceived an online physical activity intervention. Parents recognized the intervention as a useful source of motivation to help their children exercise and as a social support source. Despite the positive subjective results, researchers did not measure FMS of the children with ASD. Additionally, CHASE only provided motivation and social support, and it did not provide parents equipment or direct instruction (e.g., lesson plans) to promote FMS.

The literature suggests that parental support may be a facilitator for FMS, yet how parents can be involved in such interventions is not clear. Despite the benefits of FMS acquisition for children with ASD, interventions addressing the FMS of children with ASD, particularly parents as facilitators, are scarce [5,14]. Feasibility studies are used to understand the effectiveness of parent-mediated FMS interventions and highlight the intervention elements that contribute to FMS’s improvement in children with ASD. Hence, in this study, we report on the design and feasibility assessment of the Fit Families Program], an intervention designed to teach parents of children with ASD how to teach FMS to their children. The feasibility assessment consists of trial design, trial feasibility, and intervention feasibility indicators, including recruitment, retention, practicality of group division methods, attendance, adherence, safety, acceptability, and assessment of potential effects on FMS performance. The purpose of this pilot study was to examine the feasibility of a parent-led FMS intervention with two groups, a home-based group and an in-person workshop group, on the acquisition of FMS of children with ASD. We hypothesize that it will be feasible to implement this intervention, and we also hypothesize that both conditions will be well accepted by participants. Lastly, we hypothesize that both interventions conditions will show improvements in FMS performance.

## 2. Materials and Methods

### 2.1. Trial Design

We conducted a parallel-group randomized trial with two intervention groups in which both children and parents were participants. We used equal randomization of 1:1 to divide participants into one of two conditions, workshop group (*n* = 8) or home-based group (*n* = 7).

### 2.2. Participants

**Figure 1:**
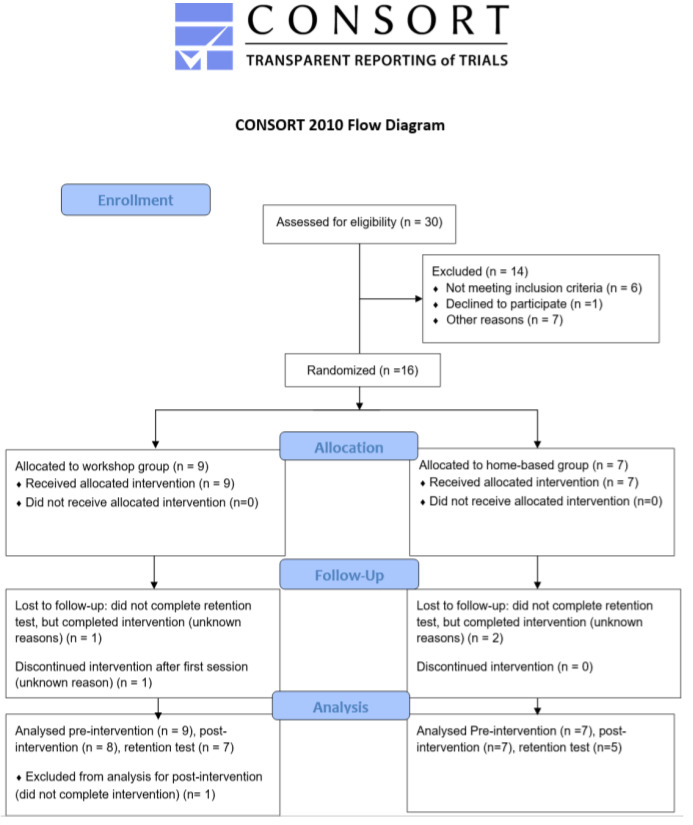
Consort Reporting of Trails Diagram.

Before the selection of participants, ethical approval was received from a University Research Ethics Board (Protocol Number: 15-246). We recruited eligible families of children with ASD from a local association supporting children with ASD that serves students and their families from several school districts in the Eastern United States. Thirty families were initially assessed, and 16 families chose to participate. All guardians provided written consent prior to their enrollment in the study (See Figure 1).

We used purposive sampling to identify possible participants for the study [20]. Children participating in the study were receiving educational services under an ASD diagnosis. We collected current and Lifetime scores on the Social Communication Questionnaire [21] to ensure that participants had a developmental history and current behavior indicative of an ASD diagnosis (See Table 1). Focusing on children aged 4–11 years captured a period where children are developing and beginning to master their FMS [4]. The inclusion criteria for parents in the study were (1) being the primary guardian of a child with an ASD diagnosis between the ages of 4 and 11 years, (2) understanding English language (spoken and written), and (3) being willing to be a participant in the intervention. Inclusion criteria for the children included (1) having a diagnosis of ASD, (2) being between the ages 4 and 11 years, (3) being able to walk independently, and (4) the child did not exhibit aggressive behaviors during game play. Participants (parents or children) who did not meet the inclusion criteria were excluded from the study.

### 2.3. Intervention Description: Fit Families Program

This study was designed to determine the feasibility of delivering a parent-mediated intervention. We randomly assigned children and parents to two different groups based on two different modalities (workshop group and home-based group) as part of a 10-week FMS intervention, known as Fit Families Program. The rationale for comparing these two groups was to explore the feasibility of parent-led interventions. Secondarily, we wanted to explore if a parent-led intervention was effective with and without in-person instruction. The intervention was grounded in the Theory of Planned Behavior (TpB) [22] and focused on physical activity topics chosen to meet the needs of families of children with ASD (e.g., sensory integration). According to TpB, expectations and values about performing a target behavior (e.g., engaging with their child with ASD in FMS) are dependent on attitudes, subjective norms, and perceived behavioral control (PBC) [22,23]. As part of this intervention, we focused on the constructs of attitudes and PBC. By focusing on these constructs, we attempted to influence parental intentions to participate in the program with their children, which consequently would influence the acquisition of FMS of the children as a result of their participation [23].

**Figure 2:**
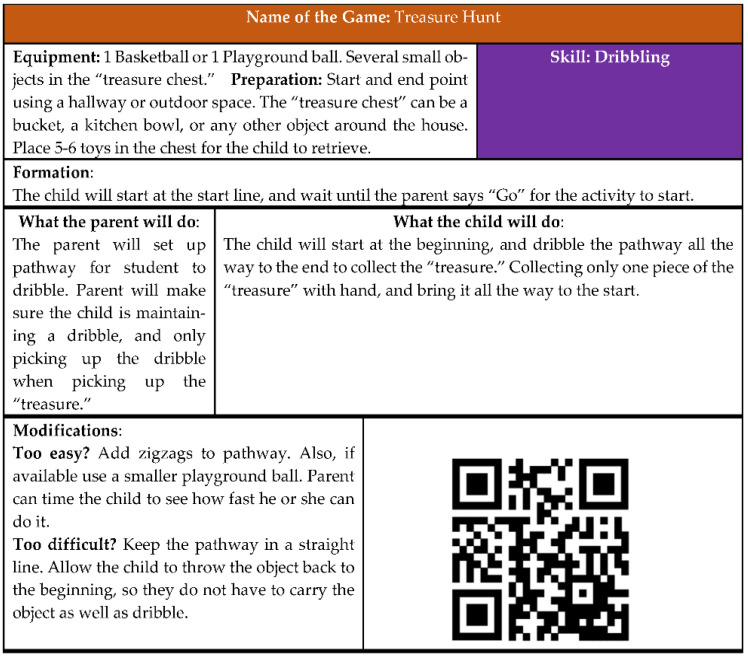
Example of one of the games included in the activity booklets.

Families in both the home-based and workshop groups were given activity booklets and adapted equipment. The intent of the activity booklet was to provide parents with a written curriculum with instructions on equipment preparation and formation, what the parent and child needed to do, modifications to make the game easier or more difficult depending on the age or ability levels of the children, and communication strategies. Each activity booklet incorporated 3–4 FMS into 20–25 games and activities designed to meet the needs of children with ASD aged 4 to 11. Each booklet was also embedded with Quick Response (QR) codes that provided direct video skill demonstration (Figure 2). The purpose of the QR codes was for parents to be able to show their child the game or activity in a video format.

Each family also received no-cost adapted equipment, which corresponded to one of the four specific topics and was tailored to the child’s age (e.g., smaller size playground ball for 4–5-year-olds) and sensory needs (e.g., slow motion soccer balls). The purpose of the adapted equipment was to encourage families to practice the FMS skills and engage in physical activity in their home and community.

In addition to the booklets and the equipment, all families received three weekly motivational text messages as reminders to stay active. We also instructed families to complete activity logs attached to the booklets. For each activity log, parents provided information about the time they spent playing the games in the activity booklet.

### 2.4. Home-Based Group

We scheduled individual in-person visits with the home-based group every 2–3 weeks to participate in a short 10–15-min interview regarding the activities’ effectiveness and quality which was used to determine program acceptance and feasibility. Families also collected the equipment and activity booklets based on the related topic (e.g., communication). We instructed families to practice the activities at home using the activity booklet and record their activities using the log.

### 2.5. Workshop Group

The purpose of the workshop group was to provide families support from physical activity professionals, provide more opportunities to practice FMS, and receive specific feedback. While the families in the home-based group only received the equipment and the activity booklets, the workshop group participated in four workshops based on the four different activity booklet topics (i.e., sensory-motor integration, communication, aquatics, and physical activity/sport). Each in-person workshop lasted approximately three hours and was divided into two parts, an educational and an active learning session (See Table 2). These workshops were held at a university setting (gymnasium and classrooms), in which all parents in the workshop group participated together during the activities.

The first part of the workshop included a parent-only educational session in a lecture style led by physical activity professionals. During this session, participants engaged in interactive discussions with the professionals delivering the workshops and other parents who shared similar experiences. For instance, during the sensory integration workshop, parents learned about sensory issues commonly associated with ASD and covered the skills of kicking, sliding, dribbling, and balance. As part of the communication workshop, parents learned about the importance of promoting and practicing communication skills by learning the underhand throw, overhand throw, catching, one hand strike, and fitness (strength and flexibility). During the third workshop, parents learned about water safety, aquatic-related activities based on the book by LePore et al. [24], and teaching strategies related to running, galloping, and skipping. During the fourth workshop, parents learned about strategies to promote physical activity and sport for their children and learned the skills of the horizontal jump, hopping, leaping, and two hand strike. Importantly, during all workshops, the role of the physical activity educator was to teach and reinforce strategies on how to interact with their children’s physical education teacher about the skills they learned.

While parents were attending the workshops, their child practiced motor skills led by university students at a ratio of two students to one child. During the first part of the workshops, university students facilitated and led individual and group games based on the activity booklet with the purpose of practicing FMS. Other research team members with an adapted physical activity background were present to support the university students and the children as needed. The second part of the workshop was an active learning session for parents and their children, in which parents applied and practiced the strategies they learned in the educational session together with their children. The parents played some of the games with their children included in the booklet. Each family was accompanied by one university student who provided feedback on how to play the game with the child. This format allowed parents to more fully comprehend the skills presented and provided additional time to practice those skills with their children. The active learning sessions lasted for approximately 30 min and were supervised by our research team to assist with family interactions to provide feedback as needed.

### 2.6. Trial Feasibility

The primary outcomes of the trial feasibility included: (a) recruitment and retention, (b) practicality of methods to divide participants into groups, and (c) fundamental motor skills data collection.

### 2.7. Recruitment and Retention

The recruitment rate was calculated as the number of eligible participants and started the intervention divided by the number of participants who were assessed for eligibility. Therefore, we considered a recruitment rate of at least 50% to be feasible as this recruitment rate was comparable to a previous parent-mediated intervention for children with ASD [25]. The retention rate was defined as the number of individuals who remained in the study until the follow-up test (3 months after intervention) as a proportion of the total number of participants assessed for eligibility. Retention rates have ranged from 76–95% in comparable parent-mediated feasibility studies [25,26]. A retention rate of at least 76% was considered an acceptable measure of trial feasibility.

### 2.8. Practicality of Group Division

Children were stratified based upon age into two groups 4–7 years old and 8–11 years old. The ages were divided based upon the hypothetical proficiency barrier, which is set at age 7 [27]. We equally distributed the number of participants for each group. Then, we randomly assigned children from either stratum to either the workshop or home group. The randomization process was conducted by computer-generated random numbers based on age and gender. Researchers did not have any decision or control in the assignment of students to a specific group. Moreover, this process was blinded, being that the personnel who completed the randomization had not interacted with the participants and were not active in the implementation of the intervention. Successful group division was determined if there was not a difference between mean ages among groups.

### 2.9. Fundamental Motor Skills Data Collection

**Figure 3:**
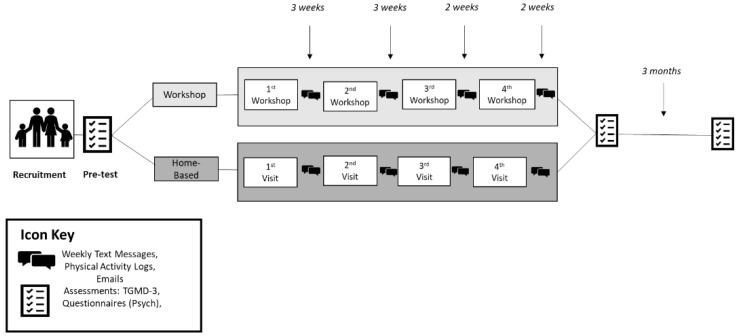
Research Design.

We used the Test of Gross Motor Development-3 (TGMD-3) [28] to assess children’s FMS. Each session was first recorded, and then the videos were analyzed. When needed, videos were watched at a slower speed. Participants were assessed at three time points: before the intervention (pre-test), after the intervention (post-test), and three months after (follow-up test). There were three weeks in between each workshop. To ensure consistency, participants were assessed three weeks after the last workshop. This was to allow participants an opportunity to practice the new skills provided during the last session (See Figure 3).

The TGMD-3 comprises six locomotor skills and seven ball skills that are developmentally appropriate for ages 3 to 10 [28]. However, this assessment has also been used with children with ASD ages 10 to 16 [29]. Locomotor skills include the run, horizontal jump, gallop, slide, skip, and hop. Ball skills include the overhand throw, underhand throw, two-hand catch, two-hand strike, one-hand strike, dribble, and kick. The TGMD-3 breaks down each skill into 3 to 5 criteria. If a criterion was met, the child received a 1; if the criterion was not met, a 0 was recorded. Each motor skill was performed and scored twice. If the child did not understand the task, the lead researcher demonstrated the skill one more time. Scores for locomotor and ball skills were obtained by adding the score from each skill in the respective subsection; an overall score was obtained by summing the scores of the two subsections. The summed total of the scores was used to find the General Motor Quotient (GMQ) that provided a single value that represents an individual’s motor ability.

### 2.10. Intervention Feasibility

The intervention feasibility was assessed through the primary outcomes of (a) attendance and adherence and (b) acceptability.

### 2.11. Attendance and Adherence

We determined intervention attendance from records documenting if families in the home group picked up their equipment every three weeks and if families in the workshop group attended the workshops. Participants were asked to complete an activity log sheet daily and submit the activity log by the end of the week. Intervention adherence was quantitatively determined by the number of activity logs families returned throughout the intervention. The activity log asked participants to detail their use of the activity booklets, including information such as games played, the number of minutes played, and comments on favorite games.

### 2.12. Acceptability

We determined intervention acceptability qualitatively via one-on-one interviews with participants pre, during (four times), and post-intervention. Interviews pre and post lasted approximately one hour. Interviews throughout the intervention lasted approximately 15 min. All interviews were audio-recorded and professionally transcribed. During the interviews, parents were asked about the information included in the activity booklet, the use of the QR codes, content and frequency of text messages, use of the adapted physical education equipment provided, and parents in the workshop group were asked about the quality of the workshops. For the purpose of this study, interview data were not analyzed looking for themes or associations. Instead, we looked for responses (positive or negative) about the questions asked.

### 2.13. Statistical Methods

Descriptive statistics were used to profile the study participants and aspects of trial and intervention feasibility. Measures of trial feasibility, specifically recruitment and retention, were calculated as a percentage using the data on the number of families assessed for eligibility, those who began the intervention, and those who completed the follow-up test. We randomized participants (children) into the two groups ensuring an equal distribution by age (4–7 and 8–11). A *t*-test was used to verify that the two groups were matched on age. After the demographic data were obtained and groups assigned, then TGMD-3 data were collected.

Even though this was not the main purpose of this study, the program effectiveness of the motor skill intervention was analyzed with a series of Wilcoxon Signed Rank Tests to identify differences in the GMQ from pre- to post-test, and post-to-follow up test for each of the groups. This was followed by a series of Mann–Whitney Test to determine if there were differences between the groups at each time point. 

## 3. Results

We recruited thirty eligible families of children with ASD from several school districts in the Eastern part of the United States. Ten families assessed for eligibility were excluded for not meeting the inclusion criteria (*n* = 6), declining to participate (*n* =1), or for another unspecified reason (*n* = 3). We stratified the remaining 20 families by age and randomly assigned them to either the workshop or home-based group. Two families who were allocated to the workshop group and three families from the home group dropped out of the study for unknown reasons before the intervention. Of the 15 families who started the study, one family from the workshop group and two families from the home-based group did not return for the follow-up test.

### Recruitment and Retention

We had a recruitment rate of 50%. Fifteen families assigned to the workshop group (*n* = 8) and home-based group (*n* = 7) completed the intervention. Twelve families completed the follow-up test, which resulted in an 80% retention rate.

- Practicality of Group Division

We randomly divided participants by age, though there was no significant difference in age between the two groups (t = 0.03, *p* > 0.98). There were also no significant differences between the intervention groups on TGMD-3 scores at pre-test (U = 22.50, *p* = 0.524).

- Intervention Attendance, Safety, and Adherence

Out of the seven home-based families, 100% of the families picked up their equipment for each session. In the workshop group, there was a 94% attendance rate. No families reported any injury occurring during the duration of the program. Ten of the 15 (67%) participants returned a completed physical activity log each week. Throughout the intervention, the average submission rate was 67% for the home group and 68% for the face-to-face group. The average number of minutes playing was calculated for each group, with the home group reporting less time playing per week (99 ± 34 min) compared to the face-to-face group (139 ± 60 min). However, the difference between groups was not significantly different (*p* < 0.05, *p* = 0.08).

- Intervention Acceptability

During the interviews, parents in both groups reported that they enjoyed the program, that they would likely participate again, and would recommend the program to other families of children with ASD. Parents expressed positive experiences with the activity booklet, physical education equipment, and text messages. This was presented from a qualitative point of view.

1. Activity Booklet

During the qualitative interviews, parents from both groups mentioned how the games and activities that were included in the activity booklets were easy to follow because of simple instructions, skill breakdowns, and supplemental QR codes. For instance, one parent in the home group stated that “It’s [the activity booklet] easy to follow. It’s got lots of tasks that are easily organized, and it breaks down simple skills and helps you figure out what you can do to work on them”. Another parent shared,

“There were some nice suggestions to make it easier or to break it down to a smaller, easier step and then as it progressed, he could elevate to what was on that reader. If it made him comfortable and it made it a lot easier for him.”

Even though the activity booklets were perceived as positive by parents, one participant, also from the home group who was a native Spanish speaker, spoke about how “It was not that by reading the booklet [my children] will not do [the activities], but for me it is not the same, it is easier to say, ‘Okay, here is the video’”. For this mother, having the video embedded as part of the activity booklet was more beneficial than having the description of the activity. Parents in the workshop group also emphasized that the variety of ways to access the activities (e.g., QR codes, written instructions) made completing the activities easier. Another participant from the workshop group also commented about the efficacy of the QR codes. She shared, “[They] were kind of like the saving grace. Some of the games were hard to visualize in my head until I saw the video.” The variety of activities and communication examples also gave children choices about completing the activities. According to one parent from the workshop group, providing multiple ways that the video can be accessed on different devices could provide parents with more choices on accessing the videos:

[My child] won’t watch the [videos] and pay attention to them. If I tried to do it on my phone he would just want to play with the phone. But if I could get it on to my computer or my TV screen, he would watch with me.

2. Adapted Physical Education Equipment

Families commented on the importance of receiving the equipment paired with the activity booklets. One parent from the home group expressed that “it’s been great to be able to receive the equipment and keep using it.” The versatility of the equipment helped the families create new activities that they would have not thought of otherwise. For example, one parent from the workshop group explained how the equipment allowed for them to break down the skill by providing visual cues, “the equipment is a huge plus. Some of it I never would have known that or thought of…I mean, just the floor dots in order to help him understand where he should be putting his feet.” Even though the equipment was beneficial, parents articulated that not all the equipment (e.g., bats) could be used inside the house.

3. Quality of Workshops

Parents reported that the workshops’ content was informative and helped them feel more confident in teaching FMS to their children with ASD. The format of the workshop, which included an informative lecture followed by a practical application of the strategies, was described by one parent as follows, “first in the class you learn things and that I liked the fact that you take this to the [gymnasium] and get to try it ourselves, and that was nice because we can participate, so it’s not [just] a lecture.” Another parent mentioned that this intervention provided a safe place for the children while parents learned strategies and connected with other parents, “I liked to be in a space and talk to other adults and listen to a presentation without worrying about what our kids were doing because our kids were downstairs having fun playing in a gym.” However, some parents shared that the workshops’ duration (3 h) was too long for their child.

4. Text Message Acceptability

During the interviews, parents indicated that the text messages were excellent reminders of the requirements of the program and were good motivational tools. Parents appreciated receiving text messages in the middle of the week. One parent from the workshop group mentioned, “I’m kind of forgetful with things. Having a little text like, ‘Oh, how are you doing through the middle of the week’, is a good reminder to keep going.” For some parents, text messages were preferred over phone calls. Another parent from the workshop group said, “the problem is that I work, so I cannot always answer phone calls. I think, for me, text messages or emails are much easier.”

- Fundamental Motor Skills Performance

There was not a significant change in their GMQ from pre-test (Median, Mdn = 66.5) to post-test (Mdn = 79) scores (Z = −0.771, *p* = 0.441, r = 0.19) for the workshop group. Those individuals who were in the home-based group had a significant increase in the GMQ score from pre-test (Mdn= 76) to post-test (Mdn= 91) (Z = −2.410, *p* = 0.016, r = 0.64). There were no significant differences between GMQ scores from post-test to follow up in the workshop group (Mdn = 68) (Z = −0.509 *p* = 0.611, r = 0.13) or the home-based group (Mdn = 91) (Z = −0.135 *p* = 0.892, r =.04). At each time point in the study there were no significant differences between the groups GMQ scores: pre-test (U = 22.50, *p* = 0.524), post-test (U = 15.50, *p* = 0.146), and the follow-up test (U = 8.00, *p* = 0.117).

## 4. Discussion

The purpose of this study was to examine the feasibility of a FMS intervention with two groups on the acquisition of FMS of children with ASD. The results demonstrated that parents can be an integral part of the acquisition of FMS for their children with ASD. In particular, the findings indicated that it is possible to recruit and retain parents of children with ASD to participate in a parent-mediated intervention.

The recruitment and retention rate recorded in this study were within the range of recruitment rates found previously with children with ASD [29,30]. Families of children with ASD often have multiple obligations related to their children’s care, which conflict with making physical activity a priority [15,16]. The fact that we were able to recruit 50% of the eligible participants was encouraging, and we identified several strategies (e.g., text messages) for recruitment and retention. Out of the 15 families that completed our intervention, 12 families completed the follow-up test, which resulted in an 80% retention rate.

This is one of the first studies in which parents and children participated together in a FMS. We acknowledge the wide age range in the study. The reason for that was that we wanted to assess the feasibility of recruiting families as a unit. We believed that if we were restricting the age range to a smaller number (e.g., 4–7 years) we would not have had enough participants. The main purpose of this study was not to test the impact of the intervention on the FMS. The main purpose was to assess the feasibility of conducting such a study. A lesson learned during the recruitment process is that first, researchers need to earn the trust of the participants and make connections with different organizations that provide services to these families.

We determined the division of participants to each condition by age. This division resulted in a non-significant difference by groups at pre-test on GMQ scores. Even though we did not find a significant difference at baseline, the GMQ scores of the home group were higher than the workshop group. Therefore, in addition to age, future studies should consider the level of GMQ and ASD severity level based. Considering GMQ and ASD symptoms is especially important since researchers have demonstrated that more ASD symptoms correlate with less developed FMS [3]. Dividing the groups only by age and not including the severity of ASD (Adaptive Behavior Assessment System, ABAS II scores) and GMQ scores may compromise creating randomized groups with an equal distribution of skills.

The home group had a 100% attendance rate, while the workshop group had a 94% attendance rate. This is a higher rate than previous studies that ranged from 80–86% [25,26]. In the study conducted by Manohar et al. [25], 94% of the participants attended four out of five sessions, and 86% attended all five sessions. On the other hand, our study met four times every three weeks, which may account for the higher attendance rate. Furthermore, Matheson et al.’s [26] study, which had 16 sessions held weekly, reported lower attendance rates ranging from 60% to 80%, which may be due to difficulties securing childcare. Another plausible reason for our attendance rate is that we encouraged attendance (via text message) and reminded parents that they would receive a new set of equipment, something that was unique to our study.

In our study, 67% of participants turned in the activity logs, which were used as a proxy of the program’s adherence. A recent feasibility study reported that 75% of their sample reported their physical activity levels (over four weeks) by submitting their responses via Facebook messenger [30]. A possible reason for our lower numbers for this may be because we relied on email and in-person delivery of activity logs. Follow-up studies should focus on delivering surveys in a participant-friendly online format.

This study built upon previous research by highlighting the value parents placed on the equipment and booklets provided as a modality to teach FMS to their children with ASD. In the Altunsöz et al.’s [17] study, parents could borrow equipment they could use during their intervention, along with lesson plans to practice activities at home. Our study expanded previous strategies by providing two different FMS modalities for support to the families. First, the equipment provided to each family was for them to keep. By teaching the parents how to use the equipment, with the accompanying activities, families were provided with the tools to continue practicing their children’s FMS beyond the intervention duration. Second, it has been demonstrated that visual supports [31] and video modeling [32] are effective and safe strategies for children with ASD. Parents received an activity booklet with embedded QR codes that leveraged these evidence-based strategies. The QR codes included videos that parents could share with their children to teach them each of the 13 FMS (e.g., locomotor and ball skills) and the games to promote or facilitate those skills. This strategy was effective and provided demonstrations that were safe and beneficial in retaining the FMS three months after the intervention. Lastly, it might be possible that the equipment motivated families to continue their participation and thus, became a contributing factor in the retention.

FMS are not acquired naturally and require practice and experience to be developed [4]. Our pilot study explored the feasibility of a parent-mediated motor skill intervention on the improvement and maintenance of FMS in children with ASD. The results demonstrated that from pre- to post-test, both groups increased their GMQ scores; however, these findings were only significant for the home group. Possible reasons why the intervention was only significant for the home group may be that the workshop group required a higher level of commitment from parents than the home group. In fact, attendance rates were higher for the workshop group, and activity logs submission demonstrated that the home group was more adherent as well. Depending on how ready the parent is to learn about how to teach skills to their child, the higher level of commitment required for participants in the workshop group may be a barrier to continuously engaging with their child in between workshops. In addition, the results demonstrated that participants in both groups were able to retain their GMQ scores three months after the intervention. This pilot study extended prior research by examining the effectiveness of two conditions based on intervention delivery, where parents were central to the intervention. Evidence supports that structured interventions can help develop FMS of children with ASD [5]. Results also align with prior research in that parents can support and teach FMS to their children and facilitate practice at home [17,18]. Despite the evidence indicating that parents should be included as part of interventions, parents have had limited involvement in FMS interventions for their children with ASD [5].

There are a couple of confounding factors related to the amount of time spent practicing skills. The at-home group had less reported practice with skills. However, they had higher GMQ scores at the post-test when compared to the workshop group. This may be due to the fact we used self-report measures instead of objective measures. In addition, it may be favorable to have a mechanism in place to assess how much children practiced outside of the workshop and outside of the lab (particularly between the post-test and retention test as the equipment was given to families). This may be a primary outcome of a future study.

This study had some limitations. First, it should be noted that the sample size was too small for statistical power to detect group differences between the workshop and home groups. However, the study design and analysis were consistent with the nature of feasibility studies, and it is important that these preliminary studies are conducted prior to conducting larger trials [25,26]. Second, this study was geographically limited to the beliefs of parents of children with ASD from the northeast region of the United States only. Including the voices of more participants, such as those from other regions of the United Sates, would have increased the generalizability of this study, for example, if a similar program were to be implemented in other regions of the country in which variables, such as weather and physical activity opportunities, might differ. In the future, researchers should address these issues. Lastly, this study lacks a control group which should be included for a larger study. Future studies should address this issue and possibly include a wait-list control group. This practice will allow those in the control group to benefit from the intervention.

In spite of the limitations mentioned above, the current study has some clinical implications. The outcomes of this pilot study suggest that parents can facilitate the acquisition of FMS of their children with ASD. Although these results were positive, there is a need to further identify effective interventions for FMS development in children with ASD. Furthermore, physical activity professionals, including physical educators, can take the findings of this feasibility study into consideration and share ideas on how parents can play with their children at home. Sending lesson plans with videos on how to play the games and activities discussed in the physical education curriculum is a starting point. Moreover, during the meeting with parents, teachers can share ideas of equipment and games parents of children with ASD can try with their children.

## 5. Conclusions

The results from this feasibility trial represent a promising parent-mediated program for children with ASD. Qualitative data demonstrated that the activity booklets, adapted equipment, and encouragement via text messages provided to the families helped facilitate physical activity participation among children with ASD. In addition, participants in the workshop group also perceived the intervention as acceptable and feasible. Further research with larger sample sizes is needed to determine which specific aspects of the home-based group led to greater improvements in motor skill performance so that future parent-mediated interventions for children with ASD and their family can emphasize these aspects.

## Figures and Tables

**Table 1:** Descriptive Statistics for Sample and TGMD-3 Across Intervention.

	Group 1 (Workshop) *N* = 8	Group 2 (Home-Based) *N* = 7
	Mean ± SD	Mean ± SD
Age (years)	7.25 ± 3.01	7.64 ± 2.56
SCQ-C	15.56 ± 4.80	13.29 ± 3.64
SCQ-L	21.11 ± 5.73	18.57 ± 6.13
ABAS	77.60 ± 15.33	84.5 ± 14.00
GAC	77.60 ± 15.33	84.5 ± 14.00
Conceptual	78.43 ± 15.90	86.67 ± 15.50
Social	79.14 ± 12.30	82.83 ± 10.90
Practical	80.00 ± 16.60	87.70 ± 14.10
Activity Log (minutes)	138 ± 60	99 ± 34
TGMD 3 (GMQ)		
Pre-test	70.38 ± 17.10	75.86 ± 13.26
Post-test	74.50 ± 11.64	85.29 ± 12.54
Retention Test	71.14 ± 16.30 *	86.40 ± 12.10 **

* *N* = 7, ** *N* = 5.

**Table 2:** Description of Intervention Topics.

Topic	Duration	Topic Description	Equipment Received	Skills Practiced
Sensorimotor	3 weeks	To learn about activities that address sensory issues associated with ASD	Spooner board Soccer Balls Basketballs (playground balls)	Kick Slide Dribble Balance
Communication	3 weeks	To learn strategies on how physical activity can be an opportunity to promote communication	Blue Disk Gator Balls Tennis Balls Hula Hoop Rackets Balloons	Underhand throw Overhand throw Catch One-hand strike Fitness: Strength and Flexibility
Aquatic	2 weeks	To learn about water safety, basic aquatic skills, and have opportunities to engage in locomotor skills at home	Aquatic Book Pool noodles Goggles Kickboards Submergible toys	Aquatic Related Skills Skip Gallop Run
Physical Activity/ Sport	2 weeks	To learn about strategies to promote physical activity and sport for their children at home and in the community	Stomp Rocket Slow-motion sand ball Batt with ball	Horizontal Jump Leap Hop Two-hand Strike

## Data Availability

The data presented in this study are available on request from the corresponding author. The data are not publicly available due to privacy regulations.
